# Multi-Omics Alterations in Rat Kidneys upon Chronic Glyphosate Exposure

**DOI:** 10.3390/biom15101399

**Published:** 2025-10-01

**Authors:** Favour Chukwubueze, Cristian D. Guiterrez Reyes, Jesús Chávez-Reyes, Joy Solomon, Vishal Sandilya, Sarah Sahioun, Bruno A. Marichal-Cancino, Yehia Mechref

**Affiliations:** 1 Department of Chemistry and Biochemistry, Texas Tech University, Lubbock, TX 79409-1061, USA; fchukwub@ttu.edu (F.C.); cristian.d.guitierrez-reyes@ttu.edu (C.D.G.R.); joy.solomon@ttu.edu (J.S.); vishal.sandilya@ttu.edu (V.S.); ssahioun@ttu.edu (S.S.); 2 Center of Basic Sciences, Department of Physiology and Pharmacology, Universidad Autónoma de Aguascalientes, Aguascalientes CP 20131, Mexico; jesus.chavezr@edu.uaa.mx (J.C.-R.); bruno.marichal@edu.uaa.mx (B.A.M.-C.)

**Keywords:** glyphosate-based herbicide, nephrotoxicity, glycomics, proteomics, inflammation

## Abstract

Clinical studies have linked glyphosate exposure to substantial morbidity, with acute kidney injury occurring in some cases. Although the toxic effects of glyphosate-based herbicides (GBHs) have been reported in several studies, their molecular impact on renal function remains poorly understood. Given the kidney’s critical role in excretion, it is particularly susceptible to damage from xenobiotic exposure. In this study, we aim to identify *N*-glycomics and proteomics change in the kidney following chronic GBH exposure, to better understand the mechanisms behind glyphosate-induced kidney damage. Kidney tissues from female and male rats were analyzed using liquid chromatography–tandem mass spectrometry. The results revealed notable changes in the *N*-glycan composition, particularly in the fucosylated and sialofucosylated *N*-glycan types. The proteomic analysis revealed the activation of immune signaling and inflammatory pathways, including neutrophil degranulation, integrin signaling, and MHC class I antigen presentation. Transcription regulators, such as IL-6, STAT3, and NFE2L2, were upregulated, indicating a coordinated inflammatory and oxidative stress response. Sex-specific differences were apparent, with female rats exhibiting more pronounced alterations in both the *N*-glycan and protein expression profiles, suggesting a higher susceptibility to GBH-induced nephrotoxicity. These findings provide new evidence that chronic GBH exposure may trigger immune activation, inflammation, and potentially carcinogenic processes in the kidney.

## 1. Introduction

Modern agriculture has embraced glyphosate-based herbicides (GBHs) since their discovery in 1970, owing to their seemingly safe nature and significant contribution to economic benefits [1,2]. GBH contains glyphosate in its salt form as its active ingredient along with other polar surfactants, such as polyoxyethyleneamide (POEA), sulfuric acid, and phosphoric acid [3]. The glyphosate’s primary mechanism of action targets the shikimate pathway, inhibiting the activity of the 5-enol-pyruvyl-shikimate-3-phosphate synthase (EPSPS) enzyme. The absence of the shikimate pathway in humans and animals lead to GBH safety margins being orders of magnitude higher than other toxic herbicides [2,4].

Over the years, the rapid rise in GBH use has increased the concern for its possible toxicity in human health. Glyphosate, being water soluble, has been detected in various water sources in Europe and the US [5]. Similarly, most crops treated with GBHs, such as soybean, corn, and wheat, have been found to have high glyphosate residues, resulting in human diet-dependent glyphosate exposure [6]. Annually, over 4000 cases of GBH exposure are reported, with around 800 requiring clinical attention [7]. Combined data from Asian countries show a case fatality rate of 7.7% from glyphosate exposure, with around 20% resulting from accidental ingestion [7,8,9,10]. Additionally, glyphosate and its secondary metabolite, aminomethylphosphonic acid (AMPA), have also been detected in urine [11] and human biofluids [12,13], confirming its accumulation in the human body. Recent findings revealed that glyphosate is detected in the urine of nearly 81% of certain population in the United States, highlighting widespread exposure [10]. The International Agency for Research on Cancer (IARC) 2015 reported the possible carcinogenicity of glyphosate in humans [14]. This has been validated by many studies using both in vitro and in vivo models [1]. Similarly, glyphosate has been reported to be genotoxic [15,16], neurotoxic [17,18,19], and nephrotoxic [20] and can lead to endocrine disruption [21] and oxidative stress [22].

The kidney, a key organ in the filtration and excretion of toxins and exogenous molecules from the body, is susceptible to potential damage from GBH exposure. A recent study [23] reported alterations in the kidney transcriptome following the consumption of a low level of a GBH formulation. The changes in gene expressions observed were associated with apoptosis via mitochondrial membrane dysfunction, necrosis, ischemia, steatosis, and fibrosis. Sub-lethal exposure to GBH has been linked to increased oxidative stress and disruption in the oxidant–antioxidant balance in the kidney [22]. Several studies associate glyphosate exposure with urinary oxidative stress and chronic kidney disease (CKD), including cases of CKD of an unknown etiology among agricultural workers [24,25]. In several studies, oxidative stress has also been shown to play a role in stimulating chronic kidney disease [26,27]. Although significant progress has been made in nephrology practice and nephrology nursing competence, there remains a growing burden of chronic kidney disease globally [28], with a limited understanding of the molecular mechanism behind the environmentally induced renal damage.

An omics analysis involves using highly sensitive technologies to study an entire system to understand molecular interactions within a biological system. Omics encompasses several subfields such as transcriptomics, proteomics, glycomics, glycoproteomics, metabolomics, lipidomics, etc. Changes in different subfields have been implicated in various diseases, suggesting the possible biomarkers of these diseases [29,30,31,32]. In a biological system, the genes produced within the nucleus of the cell are transcribed to the messenger RNA, which encodes the proteins. Most encoded proteins undergo different post-translational modifications, such as glycosylation, phosphorylation, acetylation, and methylation [33]. Protein glycosylation is an enzymatic process by which sugar molecules known as glycans are attached to proteins. This post-translational modification, present in over 50% of mammalian proteins [34], is essential for regulating both the biological properties of protein (including cell signaling, cell adhesion, and immune response) as well as the physical properties (such as, protein structure, folding, and stability) [35,36].

Protein glycosylation is commonly classified into two types: *N*-linked and *O*-linked glycosylation. The *N*-linked glycosylation occurs when the glycans are attached to an asparagine amino acid residue within the consensus sequence Asn-X-Ser/Thr (X can be any other amino acid but proline). *O*-linked glycosylation occurs when the glycans are attached to serine or threonine amino acid residues [37]. The changes in the glycan expression can result from alterations in the expression levels of certain glycosylated proteins that preferentially exhibit that specific glycan structure, an increased occupancy of glycosylation sites on non-regulated proteins, or modifications in the cellular processes or enzymatic activities that promote the formation of the *N*-glycan structure [38]. Changes in *N*-glycans can alter the function of associated proteins across several pathways [39]. These alterations in proteins and glycan expression can be influenced by diseases [40,41] and factors like environmental toxins, diet, and medications [42]. In clinical and preclinical research, proteomics analysis and glycoprofiling are increasingly being applied to uncover biomarkers of pathological conditions [41,43]. Proteins and glycans are also known to be instrumental in early diagnostics and prognostics of several diseases [44,45] and environmental toxicology [46]. We recently reported a proteomics study that revealed a significant increase in neuroinflammation following chronic exposure to organophosphate pesticides [42]. Similarly, we have reported several alterations in the metabolites and *N*-glycans associated with immune responses and neuroinflammation in the serum, prefrontal cortex, and hippocampus of rat models, suggesting possible impaired memory and cognitive function after exposure to GBH [4,18,19].

This study, however, presents changes observed in the *N*-glycan profile and protein expressions in kidney tissues of rats exposed to glyphosate-based herbicides using a liquid chromatography–tandem mass spectrometry (LC-MS/MS) approach. The LC-MS/MS technique has gained prominence in the omics field due to its exceptional sensitivity, resolution, and precision, which allow for a highly sensitive analysis, structural elucidation, and quantification [29]. The primary aim of this study is to explore the molecular alterations in the kidney following chronic exposure to a glyphosate-based herbicide, with a focus on the *N*-glycan composition and proteomic expression. The secondary aim includes identifying key biological pathways involved in GBH-induced renal dysfunction, assessing sex-specific differences in response to exposure, and uncovering potential biomarkers associated with GBH-induced nephrotoxicity.

## 2. Materials and Methods

### 2.1. Materials and Reagent

Ammonium bicarbonate (ABC), borane-ammonia, dithiothreitol (DTT), dimethyl sulfoxide (DMSO), iodoacetamide (IAA), iodomethane, sodium deoxycholate (SDC), and sodium hydroxide (NaOH) beads were obtained from Sigma-Aldrich (St. Louis, MO, USA). Acetic acid, acetonitrile (ACN), formic acid, high-performance liquid chromatography (HPLC)-grade water, and methanol were purchased from Fisher Scientific (Fair Lawn, NJ, USA). The peptide-*N*-glycosidase F (PNGase F) enzyme was purchased from New England Biolabs (Ipswich, MA, USA), while the Trypsin/Lys-C mix MS-grade was obtained from Promega (Madison, WI, USA). The molecular-biology-grade zirconium beads were acquired from OPS Diagnostics (LLC, Lebanon, NJ, USA). The GBH used for this study was the Rival^®^ herbicide (glyphosate concentration of 680 g per kilogram) purchased from Monsanto (St. Louis, MO, USA).

### 2.2. Animal Study

**Figure 1:**
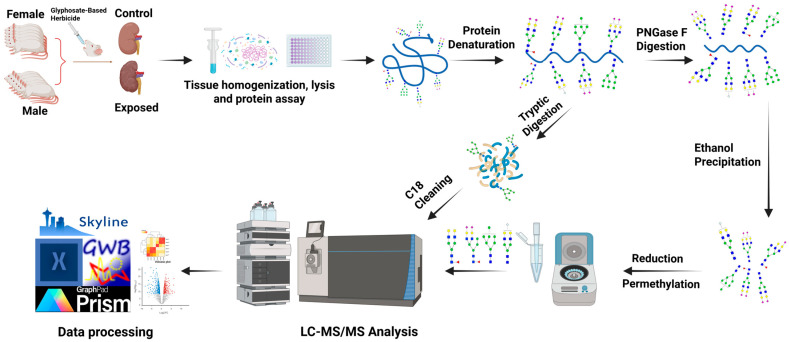
Experimental workflow for the multi-omics analysis. The glycans were purified, reduced, and permethylated prior to LC-MS/MS analysis. The digested peptides were subjected to C18 cleaning prior to LC-MS/MS analysis). *N*-Glycan structures symbols are denoted as GlcNAc (*N*-acetylglucosamine) 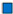, Gal (galactose) 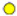, Man (mannose) 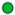, Fuc (fucose) 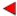, NeuAc (*N*-acetylneuraminic acid) 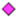, and NeuGc (*N*-glycolylneuraminic acid) 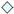.

This study was reported in accordance with the ARRIVE 2.0 guidelines [47], and the completed checklist is provided in the Supplementary Information. Twenty-five Sprague Dawley rats obtained from the Institutional Vivarium of the Autonomous University of Aguascalientes were utilized for this study. The rats consisted of two cohorts: females (*n* = 13) and males (*n* = 12) between postnatal days 22 and 24. The National Research Council Guide for Care and Use of Laboratory Animals [48] and the Mexican Guideline for Animal Care NOM-062-ZOO-1999 experimental procedures were adhered to when conducting this experiment. The number of rats for this study was selected following previously reported guidelines [49]. The rats were kept in a controlled environment with a temperature between 20 °C and 22 °C, humidity at 45 to 55%, 12 h light/dark cycle, and free access to food and water. The rats were habituated to test environment for 7 days before the group allocation using manual simple randomization to form the experimental groups as follows. Six out of thirteen female rats and six out of twelve male rats were assigned to the control group, while seven other female rats and six other male rats were assigned to the glyphosate-based-herbicide-exposed group. The animals receiving the same treatment were kept in groups of 3 to 4 rats per cage. The control groups of the different cohorts were given oral gavages of water daily at a dose of 1 mL per kilogram of bodyweight, while the GBH-exposed groups were administered GBH solutions orally through gavage at a dose of 100 mg per kilogram bodyweight per day for 12 weeks. The dosages were based on a similar study [50]. Preliminary behavioral and cognitive tests were further completed in the studied cohorts and reported in our previous study [18]. Afterward, euthanasia was performed with an overdose of sodium pentobarbital (via intraperitonial injection), and the rats’ kidneys were harvested and stored at −80 °C. Subsequently, LC-MS/MS glycomics and proteomics analyses were conducted on the kidney tissues. The step-by-step experimental workflow for the analyses is illustrated in Figure 1.

### 2.3. Tissue Homogenization, Lysis, and Protein Extraction

The harvested kidney tissue samples were carefully placed in a Dounce homogenizer and crushed to achieve smooth consistency. To lyse the tissue cells, aliquots of 100 µL of 50 mM ABC buffer were added to ~1 mg of the samples and then transferred to a 2 mL microcentrifuge tube containing 100 mg of 400 µm zirconium beads. Then, 100 µL of 5% SDC solution was also added to the tubes, and the samples were further homogenized using a Beadbug microtube (Benchmark Scientific, Edison, NJ, USA) at 4000 rpm for 30 s (at 4 °C). After three cycles of the process (with a 30 s break in between), the lysates were sonicated on ice for an hour. The samples were centrifuged at 14,800 rpm for 10 min, and the supernatant was collected in an Eppendrof tube. A bicinchoninic acid (BCA) protein assay kit (Thermo Sci.) was used to determine the protein concentration of the samples.

### 2.4. N-Glycan Release, Purification, Reduction, and Permethylation

About 10 µg of protein from each sample was denatured at 90 °C for 15 min and allowed to cool. Afterward, 500 U of PNGase F enzyme was added to the samples followed by incubation at 37 °C. After 18 h, 90% ethanol was introduced, and the samples were frozen at −20 °C for 30 min to precipitate the proteins. The samples were centrifuged at 14,800 rpm for 10 min. The supernatant containing *N*-glycans was transferred to a new sample tube and dried. To reduce the purified glycan, 10 mg/mL solution of borane–ammonia complex in water was prepared, and 10 µL of the solution was added to the samples. The samples were incubated at 60 °C for an hour. Subsequently, the borane residue in the samples was removed by adding 1000 µL of methanol to form methyl borate. This process was repeated five times until all the formed methyl borate had evaporated completely, leaving the samples dry. The reduced *N*-glycans underwent solid-phase permethylation as previously described by Kang et al. [51]. The microspin column containing NaOH beads suspended in DMSO was conditioned by washing with 200 µL of DMSO and centrifuging at 1800 rpm for 2 min. The dried samples were resuspended in 30 µL of DMSO, 20 µL of iodomethane, and 1.2 µL of water. The sample solution was transferred to the column and incubated in the dark at room temperature. After 25 min, 20 µL of iodomethane was added to the column and incubated for an additional 15 min. The column was then centrifuged at 1800 rpm for 2 min to collect the permethylated *N*-glycans. The column was further rinsed using 30 µL of ACN to ensure complete collection of the eluent. The samples were dried and resuspended in 20% ACN and 0.1% formic acid for LC-MS/MS analysis.

### 2.5. Tryptic Digestion

About 20 ug of proteins from the lysed kidney tissue stock was transferred to a new Eppendorf tube. The proteins were denatured at 90 °C for 15 min. After cooling, 1.25 µL of 200 mM dithiothreitol (DTT) was added to reduce the disulfide bond linkages in the proteins. The samples were incubated at 60 °C for 45 min. Next, 5 µL of iodoacetamide (IAA) was added to the samples and incubated at 37 °C for another 45 min. Following incubation, an additional 1.25 µL of DTT was added to the sample and incubated at 37 °C to quench the alkylation reaction. After the 30 min incubation, the trypsin enzyme was added to the sample in the ratio of 1:25 (enzyme: sample protein mass) and incubated at 37 °C for 18 h. Afterward, the enzyme activity was quenched using 1 µL of formic acid, and the samples were dried using a speedVac concentrator.

### 2.6. C18 Desalting

The binding solution (0.1% formic acid), releasing solution 1 (60% ACN and 0.1% formic acid), and releasing solution 2 (100% ACN) for the C18 desalting process were first prepared. The microspin column, filled with C18 materials suspended in ACN, was conditioned by adding 50 µL of releasing solution 1 and centrifuging at 1000 rpm for 1 min. This process was repeated three times followed by adding 50 µL of the binding solution and centrifuging at the same speed for 1 min (3 times). The samples were resuspended in 100 µL of the binding solution. The sample solution was transferred to the column and centrifuged at 1000 rpm for 2 min before incubating. After 2 min of incubation, the flow through was applied to the column again and centrifuged at the same speed for another 2 min. This process was repeated three times to ensure complete binding of the peptide to the C18 material. Then 50 µL of the binding solution was used to wash out salts from the samples. To retrieve the pure peptides, 50 µL of releasing solution 1 was first applied to the column and centrifuged at 1000 rpm for 2 min. The process was performed three times before the subsequent addition of releasing solution 2 and centrifuging at the same speed for 2 min (repeated 3 times). The eluents were collected, dried, and resuspended in 2% ACN and 0.1% formic acid for LC-MS/MS analysis.

### 2.7. LC-MS/MS Glycomics Analysis

The permethylated *N*-glycans were analyzed using an Ultimate 3000 nanoUHPLC system (Thermo Scientific, San Jose, CA, USA) coupled to an Orbitrap Fusion Lumos Tribrid Mass Spectrometer (Thermo Scientific, San Jose, CA, USA) with a nanoESI source. About 2 µg of the sample was injected into the instrument and further purified online using a C18 Acclaim PepMap trap (75 µm × 2 mm, 3 µm, 100 Å, Thermo Scientific). An Acclaim PepMap C18 capillary column (75 µm × 15 cm, 2 µm, 100 Å, Thermo Scientific) was used to separate the *N*-glycan sample at a temperature of 55 °C and a 0.35 µL/min flowrate. A 60 min chromatographic gradient was used with mobile phase A (98% H_2_O, 2% ACN and 0.1% FA) and mobile phase B (100% ACN and 0.1% F.A). For the gradient, 20% mobile phase B was used in the first 10 min. For the next one minute, it was increased to 42%. Subsequently, mobile phase B increased gradually to 55% from 11 min to 48 min of the runtime. It was further increased to 90% at 49 min of the run and was kept constant for the next 5 min before returning to the initial 20% to re-equilibrate the column. For the MS identification, positive ion mode was used, with a spray voltage of 1.6 kV and a capillary temperature of 305 °C. The full MS covered a mass range from 400 to 2000 *m*/*z* for detecting the precursor ion with a mass resolution of 120,000. The data-dependent acquisition (DDA) mode was used for the MS/MS scan. The top 20 precursor ions with the highest intensity in the full MS scan were selected for collision-induced dissociation (CID) fragmentation. The activation time was set at 10 ms and a normalized collision energy of 35%. The MS/MS scan was set at a resolution of 30,000 with AGC target of 5 × 10^4^.

### 2.8. LC-MS/MS Proteomics Analysis

The LC-MS/MS proteomics analysis used Dionex 3000 Ultimate nanoLC system (Thermo Sci., Sunnyvale, CA, USA) coupled with a QExactive HF Mass Spectrometer (Thermo Sci., San Jose, CA, USA). About 2 ug of the sample was injected into the nanoLC system at a flow rate of 0.35 µL/min and purified using a C18 Acclaim PepMap trap (75 µm × 2 mm, 3 µm, 100 Å, Thermo Scientific). The samples were separated on the Acclaim PepMap C18 capillary column (75 µm × 15 cm, 2 µm, 100 Å, Thermo Scientific) at a temperature of 30 °C. A 120 min chromatographic gradient with mobile phase A (98% H_2_O, 2% ACN and 0.1% FA) and mobile phase B (98% ACN, 2% H_2_O and 0.1% FA) was used to separate the peptides. The gradients are as follows: 5% mobile phase B was used for the first 10 min and increased to 35% for the next 70 min. The mobile phase B was further increased to 60% between 80 min and 110 min of the runtime. Within the next 13 min, it was again increased to 90% and kept constant for 5 min before returning to the initial 5% to re-equilibrate the column. The acquisition in the mass spectrometer was performed in positive ion mode with a mass range of 400 to 2000 *m*/*z* at a 120,000 mass resolution (full scan). The top precursor ions were selected for high-energy collision dissociation (HCD) fragmentation. The data-dependent acquisition mode was used for the MS/MS scan with an AGC target of 5 × 10^4^ and a resolution of 60,000.

### 2.9. Data Analysis

For the data analysis, raw files obtained from the LC-MS/MS glycomics analysis were manually annotated using Xcalibur 4.2 software (Thermo Scientific) to identify all the *N*-glycan present in the samples. The absolute abundance of the identified glycan species was quantified using Skyline software version 21.2.0.536 (MacCoss Lab, University of Washington, Seattle, WA, USA). The absolute abundance of the *N*-glycans was normalized by dividing each *N*-glycan abundance by the total abundance of all the glycans. Unsupervised principal component analysis (PCA) was performed on the results, followed by statistical analysis. Statistical analysis using the Welch *t*-test was performed, and the *p*-value cut-off was set as 0.05. *N*-glycans with *p*-values less than 0.05 were considered to be statistically significant.

For the proteomics study, the raw files from LC-MS/MS proteomics analysis were processed using Proteome Discoverer software version 2.5 (Thermo Scientific, Bremen, Germany). The protein identification and quantification were performed on the software using the UniProtKB/Swiss-Prot rat protein database. Since trypsin, which is specific in R and K cleavage, was used, the missed cleavage was set as two. A minimum of six amino acids were required for the identified peptides. Acetylation of *N*-terminus and methionine oxidation were set as variable modifications, while carbamidomethylation of cysteine was set as a constant modification. The criteria for identification were set with 95% confidence intervals and a 1% false discovery rate using the target–decoy method. Additional data analysis was conducted using R (v4.4.2), which included relative normalization, unsupervised principal component analysis (PCA), differential expression analysis, and figure creation. Welch’s T-test was used for pairwise comparisons, and proteins were deemed differentially expressed if their *p*-value was less than 0.05. Ingenuity Pathway Analysis (IPA) software version 107193442 by Qiagen was employed for further pathway analysis. Only those proteins that were differentially expressed (*p*-value < 0.05) were subsequently included for IPA. For both *N*-glycomics and proteomics study, statistical analysis and figure generation were conducted in R (v4.4.2), and Xcalibur version 4.2 software (Thermo Scientific, Bremen, Germany) was used for generation of the *N*-glycan extracted ion chromatograms.

## 3. Results

### 3.1. N-Glycomics Analysis

**Figure 2:**
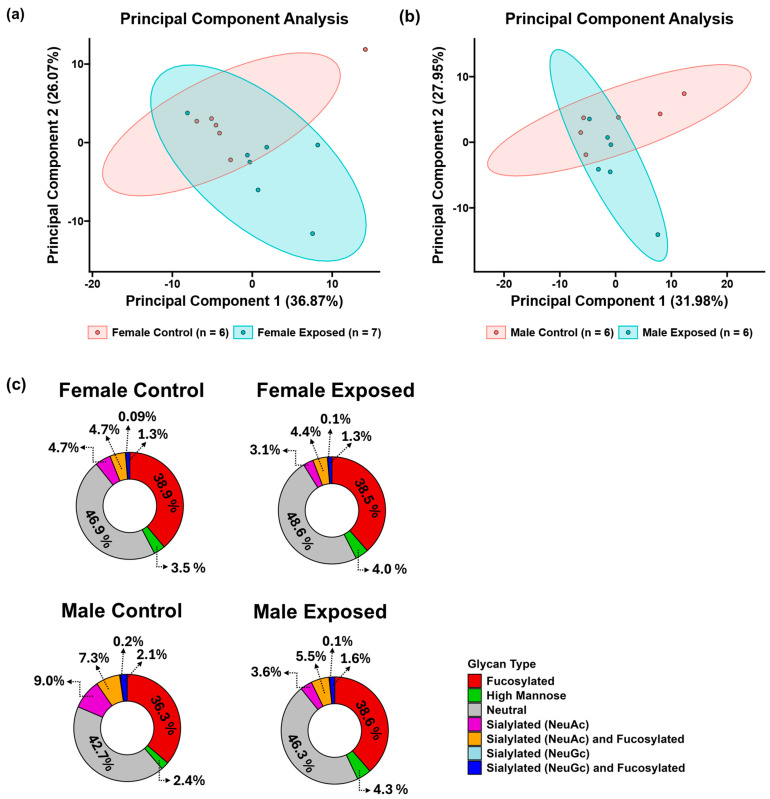
Two-dimensional unsupervised principal component analysis (PCA) with a 95% confidence level showing the glycomic clustering of the control group versus the GBH-exposed group of the (**a**) female cohort and (**b**) male cohort. (**c**) Pie chart displaying the relative abundance of fucosylated, high mannose, neutral, sialylated (NeuAc), sialylated (NeuGc), and sialofucosylated *N*-glycans. Female control (*n* = 6), female exposed (*n* = 7), male control (*n* = 6), and male exposed (*n* = 6).

A total of 117 *N*-glycans were identified and quantitatively profiled from the rat kidney tissue samples. To simplify the visualization of the *N*-glycan expression patterns, a two-dimensional unsupervised principal component analysis was performed as shown in Figure 2a,b for the female and male cohorts, respectively. The principal component analysis revealed a good separation of the control and GBH-exposed groups in the different cohorts, capturing the variance in the dataset. This allowed for the observation of *N*-glycomic changes associated with the transition from normal kidney function to a GBH-exposed state in rats. An extracted ion chromatogram of the identified *N*-glycans is shown in Figure S1. Both the MS and MS/MS scans were used for the identification of the composition and structure of the *N*-glycans, as shown in Figure S2. The *N*-glycans were designated using a five-digit code to represent their composition—such that 5-6-1-2-0 represents 5-*N*-acetyl glucosamine, 6-Hexose (galactose or mannose), 1-Fucose, 2-*N*-acetyl neuraminic acid (NeuAc), and 0-*N*-glycolylneuraminic acid (NeuGc) residues—or were designated as HexNAc_5_Hex_6_Fuc_1_NeuAc_2_.

To better understand the glycome differences, the identified *N*-glycans were classified into different types, including fucosylated, high mannose, neutral, sialylated (both NeuAc and NeuGc), and sialofucosylated *N*-glycans. As shown in Figure 2c, the fucosylated and neutral *N*-glycans were predominant in both cohorts, with relative abundances of approximately 40% and 50%, respectively. Conversely, the glycans carrying NeuGc units had the lowest abundance in both cohorts, with a relative abundance of approximately 0.1%. The high mannose, sialylated NeuAc, and sialofucosylated *N*-glycans each exhibited a relative abundance of less than 10%. This finding aligns with previous studies highlighting the sialylation and fucosylation of kidney tissues [52,53]. The Shapiro–Wilk test (Table S1) confirmed the normal distribution of the data, permitting the use of Welch’s T-test for the statistical analysis. The statistical comparison between control and GBH-exposed groups revealed twelve significantly altered *N*-glycans (*p*-value < 0.05) in the female cohort, all of which were elevated in the GBH-exposed group. In the male cohort, seven *N*-glycans exhibited significant changes (*p*-value < 0.05), with five showing a decreased abundance and two showing an increased abundance in the GBH-exposed group. Tables S2 and S3 show the relative abundance and *p*-values of the statistically significant *N*-glycans in the female cohorts and male cohorts, respectively.

**Figure 3:**
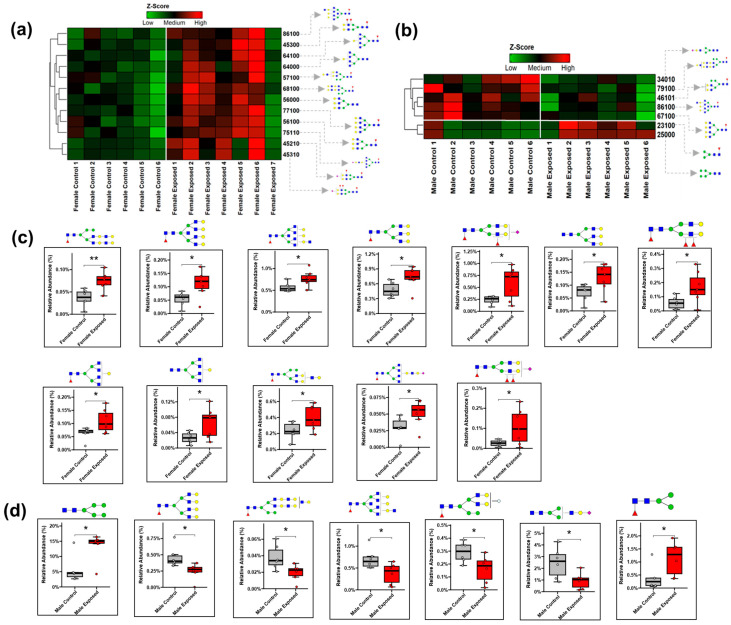
The heatmap representation of the statistically significant *N*-glycans in the (**a**) female cohorts and (**b**) male cohorts. The red color denotes *N*-glycans with increased abundance, and the green color denotes *N*-glycans with decreased abundance. The boxplots of the significant *N*-glycans in females (**c**) and males (**d**) (*—*p*-value < 0.05; **—*p*-value < 0.01). *N*-glycan symbols are as in Figure 1.

To further illustrate the expression patterns of these *N*-glycans, heatmaps and boxplots were made. Figure 3a displays the heatmap of the differential abundance of the significant *N*-glycans in the females, while Figure 3b shows that of the males. Figure 3c,d shows the boxplots of the significant *N*-glycans in the females (3c) and males (3d). Notably, ten out of the twelve significant *N*-glycans in the females were either fucosylated or sialofucosylated. In the males, five of the seven significant *N*-glycans were fucosylated. The *N*-glycan HexNAc_8_Hex_6_Fuc_1_ was significantly altered in both sexes, showing an increased abundance in the females and a decreased abundance in the males.

To assess potential trends in the abundance of *N*-glycans in the rat kidney tissues independent of sex, a combined cohort analysis was conducted, comparing all control samples vs. all GBH-exposed groups. The principal component analysis did not show much separation between the control and GBH-exposed group. The analysis revealed two significantly altered *N*-glycans. The *N*-glycan HexNAc_3_Hex_4_NeuAc_1_ showed a decreased abundance in the GBH-exposed group compared to the control, while HexNAc_4_Hex_5_Fuc_3_NeuAc_1_ showed an increased abundance (Figure S3). Notably, HexNAc_4_Hex_5_Fuc_3_NeuAc_1_ was exclusively significant in the female cohort, whereas HexNAc_3_Hex_4_NeuAc_1_ was specific to the male cohort.

### 3.2. Proteomics Analysis

**Figure 4:**
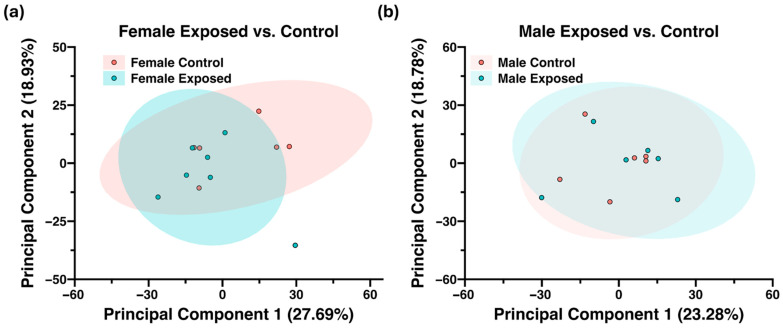
Two-dimensional unsupervised principal component analysis (PCA) with a 95% confidence level showing the proteomics clustering of the control group versus the GBH-exposed group of the (**a**) female cohort (*n*_control_ = 6; *n*_exposed_ = 7) and (**b**) male cohort (*n*_control_ = 6; *n*_exposed_ = 6).

A total of 7845 proteins were identified and quantified using the proteome discoverer software (Thermo Scientific). This comprised of 1065 high-confidence (1% FDR), 120 medium-confidence (<5% FDR), and 6660 low-confidence proteins (>5% FDR). The low-confidence proteins as well as proteins absent in 75% of the samples were filtered out. Only the 1123 high- and medium-confidence proteins were considered for further analysis. An unsupervised PCA (Figure 4a,b) revealed a more distinct separation between control and GBH-exposed groups in female cohorts compared to males.

**Figure 5:**
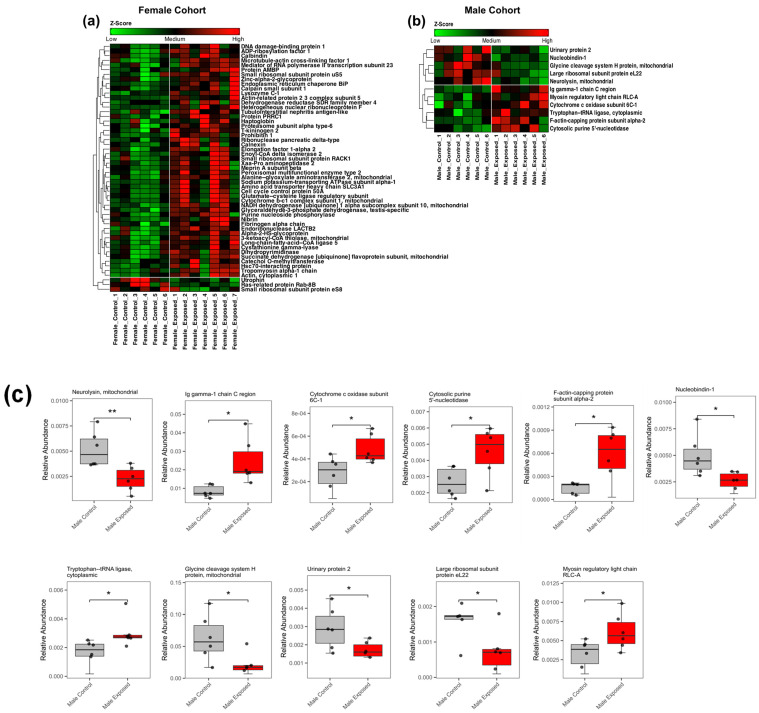
The heatmap representation of the differentially expressed proteins in the (**a**) female cohort and (**b**) male cohort. The red color denotes *N*-glycans with increased abundance, and the green color denotes (**c**) the boxplots of the statistically significant proteins in the male cohort (*—*p*-value < 0.05; **—*p*-value < 0.01).

The Shapiro–Wilk test (Table S1) confirmed the normal distribution of the data, permitting the use of Welch’s *t*-test for the statistical analysis. The statistical analysis identified fifty-three differentially expressed proteins in the female cohort, as shown in Table S4. Additionally, eleven differentially expressed proteins were identified in the male cohort, as shown in Table S5. Among the female cohort, the majority of the proteins (50 proteins) were upregulated, while three were downregulated. This trend is similar to that observed in the glycomics data. In contrast, the male group showed a more balanced distribution, with six proteins upregulated and five downregulated. The heatmaps in Figure 5a,b show the differential expression patterns of the proteins in the female and male cohorts, respectively.

In the female cohorts, the downregulated proteins, such as utrophin, Rab-8B, and eS8, were associated with protein synthesis, while many upregulated proteins were linked to tumor progression. Some known kidney damage markers, such as Alphas-2-HS-glycoprotein [54] and Haptoglobin [55], were also found to be upregulated. In the male cohort, proteins including urinary protein 2, nucleobindin-1, H-protein, large ribosomal subunit protein eL22, and neurolysin were downregulated, whereas Ig Gamma 1 chain C, myosin regulatory light chain RLC-A, Cytochrome c oxidase subunit 6C isoform 1, Tryptophan–tRNA ligase, cytoplasmic, F-actin-capping protein subunit alpha-2, and Cytosolic purine 5′-nucleotidase II were upregulated. The boxplots of these proteins are shown in Figure 5c. Interestingly, no overlapping protein was observed to be significant in both sexes, highlighting sex-specific responses to GBH exposure.

To further understand the protein expressions across sexes, a combined analysis of all control versus GBH-exposed samples was performed. This comparison revealed 39 significantly altered proteins, as depicted in Figure S4, of which 38 were upregulated and 1 was downregulated, indicating a general trend of protein upregulation following the GBH exposure across the dataset.

### 3.3. Ingenuity Pathway Analysis

**Figure 6:**
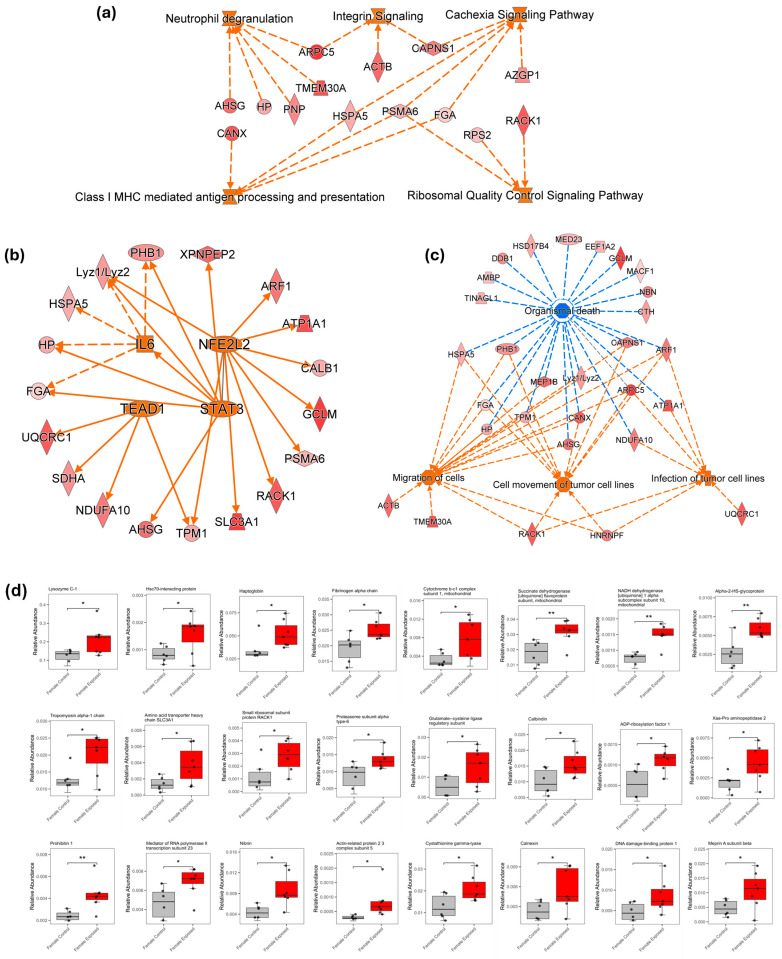
Predicted canonical pathways associated with the differentially expressed proteins in the female cohort are shown in (**a**). The activated pathways include cachexia signaling pathway, neutrophil degranulation pathway, class I MHC-mediated antigen processing and presentation pathway, integrin signaling pathway, and ribosomal quality control pathway. (**b**,**c**) present the key upstream transcription regulators and related diseases and functions identified in the female cohort. Orange color depicts activation, while blue represents inhibition. (**d**) displays boxplots highlighting several significantly altered proteins involved in the pathways, diseases, and functions in the female cohort (*—*p*-value < 0.05; **—*p*-value < 0.01).

To further understand the pathways and disease function implications of the observed proteomic changes, an ingenuity pathway analysis was performed using the significantly altered proteins. In the female group, the analysis identified some activated signaling pathways, including cachexia, integrin activity, ribosomal quality control, neutrophil degranulation, and the antigen presentation via the class I MHC (major histocompatibility complex), as depicted in Figure 6a. These pathways reflect a coordinated cellular response involving inflammation, immune responses, and tumor progression. Some transcription regulators, such as Interleukin-6 (IL-6), Signal Transducer and Activator of Transcription 3 (STAT3), nuclear factor erythroid 2–like 2 (NFE2L2), and transcriptional enhanced activated domain 1 (TEAD1), were predicted to be activated, as depicted in Figure 6b. Figure 6c shows the disease and biological functions associated with the altered proteins, such as the enhanced migration of cells and the cell movement of tumor cell lines, along with an increased susceptibility to infection. In contrast, organismal death was predicted to be inhibited, suggesting a shift toward a pro-survival, tumor-promoting environment in response to GBH exposure. Figure 6d shows the boxplots of some of the implicated proteins in the pathways, diseases, and functions in the female cohorts.

In the male cohort, however, due to the relatively small number of significantly altered proteins, the IPA did not yield any predictions regarding activated pathways or the associated disease and functions. The combined analysis of both sexes, as shown in Figure S5a, predicted the activation of transcription regulators such as activating transcription factor 4 (ATF4), cAMP response element-binding protein 1 (CREB1), and angiotensinogen (AGT), which are involved in stress responses, metabolism, and gene expression. Meanwhile, regulators such as the tumor necrosis factor were predicted to be downregulated, suggesting the potential suppression of immune and apoptotic pathways. Figure S5b,c show associated diseases and functions and the boxplots of some of the significant proteins in the combined analysis, respectively.

## 4. Discussion

Recent studies increasingly demonstrate the adverse effects of glyphosate on human health, emphasizing the need to investigate the molecular changes triggered by glyphosate exposure. Exposure to GBHs can occur in various ways, including consuming contaminated food and water, inhaling airborne particles near sprayed areas, or through skin contact during herbicide application or from environmental residues [3]. An animal study mimicking chronic exposure to glyphosate will help elucidate the molecular mechanisms of GBH nephrotoxicity, identify early biomarkers of kidney injury associated with GBHs, and provide a scientific basis for evaluating the safety of glyphosate exposure in humans.

In this study, we used high-resolution liquid chromatography and tandem mass spectrometry (LC-MS/MS) to investigate *N*-glycan and protein alterations in rat kidneys following GBH exposure. Given that glyphosate effects have been shown to be sex-specific [56,57,58], this analysis was conducted based on sex. The glycomics analysis revealed subtle yet biologically significant changes in the *N*-glycans in the kidney of GBH-exposed rats, particularly in females.

In the female rats, twelve *N*-glycans were significantly altered following chronic GBH exposure, with all showing elevated levels in the GBH-exposed group compared to the control. Among these, seven were fucosylated *N*-glycans (HexNAc_4_Hex_5_Fuc_3_, HexNAc_5_Hex_6_Fuc_1_, HexNAc_5_Hex_7_Fuc_1_, HexNAc_6_Hex_4_Fuc_1_, HexNAc_6_Hex_8_Fuc_1_, HexNAc_7_Hex_7_Fuc_1_, HexNAc_8_Hex_6_Fuc_1_) and three were sialofucosylated (HexNAc_4_Hex_5_Fuc_3_NeuAc_1_, HexNAc_4_Hex_5_Fuc_2_NeuAc_1_, HexNAc_7_Hex_5_Fuc_1_NeuAc_1_). Fucosylated *N*-glycans are known mediators of immune activation and signal transduction [59]. The abnormal elevation of the fucosylated *N*-glycans observed in the GBH-exposed group may result from dysregulated fucosyltransferase activity, altered GDP-fucose levels, or substrate availability. Aberrant fucosylation has been shown to influence the onset of inflammation and cancer [59,60], enabling tumor cells to evade immune surveillance and develop drug resistance. This would provide a convenient environment for tumors to thrive in the GBH-exposed kidneys. Some of the observed fucosylated *N*-glycans were core fucosylated *N*-glycans, which are known potential biomarkers for cancer diagnoses and contributors to renal fibrosis through cytokine receptor modulation [61,62,63]. Notably, sialofucosylated *N*-glycans—such as HexNAc_4_Hex_5_Fuc_2_NeuAc_1_ and HexNAc_4_Hex_5_Fuc_3_NeuAc_1_, which are observed to be altered in our study—have been associated with enhanced cancer cell adhesion and metastasis [64], as well as with neurodegenerative diseases such as Alzheimer’s [65]. Similarly, Drake et al. recently reported the elevated expression of fucosylated and sialofucosylated glycans in human kidney tumors [52]. This supports a possible link between GBH exposure and tumor progression. Additionally, two high mannose *N*-glycans (HexNAc_5_Hex_6_ and HexNAc_6_Hex_4_) showed an increased abundance in the female cohort. Alterations in these glycans have been linked to an enhanced metastatic potential in breast cancer models [66].

Furthermore, the male cohort exhibited fewer significantly altered *N*-glycans compared to the females. Five out of the seven significant *N*-glycans were also fucosylated (with HexNAc_2_Hex_3_Fuc_1_ showing increased abundance, and the rest showing decreased abundance in the GBH-exposed group). This suggests a similar impact of the GBH exposure on the male kidneys. The only high-mannose glycan structure found to be significantly altered was HexNAc_2_Hex_5_, which exhibited an increased abundance in the GBH-exposed group. HexNAc_2_Hex_5_ has been reported to be abundant in different mammalian tissues [67,68] and has also been observed to be elevated in cancerous tissues [66,69]. The two significantly altered sialylated *N*-glycans (HexNAc_3_Hex_4_NeuAc_1_ and HexNAc_4_Hex_6_Fuc_1_NeuGc_1_) observed in the male cohort showed a decreased abundance in the GBH-exposed group. Reduced sialylation can impair podocyte function and glomerular filtration due to the loss of the surface negative charge, thereby increasing the susceptibility to glomerular injury [70]. Interestingly, the *N*-glycan HexNAc_8_Hex_6_Fuc_1_ was significant in both cohorts but showed opposite trends in males (decreased abundance) and females (increased abundance), suggesting possible sex-specific regulatory mechanisms.

The result from the combined sex analysis showed the sialylated and sialofucosylated *N*-glycans HexNAc_3_Hex_4_NeuAc_1_ and HexNAc_4_Hex_5_Fuc_3_NeuAc_1_ to be significant, further supporting the possibility of disrupted immune responses and possible cancer-related activity in renal tissues exposed to GBH. Our findings suggest that the alterations in *N*-glycans observed in the kidney following chronic GBH exposure could influence inflammation and tumor progression in the kidney. The trends we observed in the different cohorts align with our prior findings in brain tissues [18], where female rats exhibited greater sensitivity to glycomic changes as a result of GBH exposure.

The protein expression profile of the GBH-exposed kidney tissues, particularly in the female rats, revealed a coordinated activation of pathways that collectively points to a tissue microenvironment that promotes chronic inflammation, immune dysregulation, metabolic imbalance, and, ultimately, carcinogenesis. Interestingly, 94% of the significantly altered proteins (50 proteins) in the female rat kidneys were upregulated, indicating a strong molecular response to GBH exposure. These proteins are associated with the predicted activation of the pathways shown in Figure 6a.

One of the key activated pathways is the cachexia signaling pathway, a hallmark of the systemic inflammation and metabolic dysfunction often observed in cancer and chronic kidney disease tissues [71]. Proteins involved in this pathway, such as proteasome subunit alpha type-6, calpain small subunit 1, and zinc-α2-glycoprotein, are integral to muscle catabolism, intracellular proteolysis, and energy metabolism [72]. Their increased expression in the GBH-exposed rat kidneys may reflect an early onset of renal tissue catabolism, a response commonly triggered by pro-inflammatory cytokines like TNF-α and IL-6 [73]. These inflammatory and metabolic stresses are recognized precursors to tumor development, as they promote sustained cellular stress and metabolic dysfunction [74].

Similarly, the integrin signaling pathway was also predicted to be activated. Integrins serve as critical mediators of cell–extracellular matrix (ECM) interactions, regulating cell adhesion, survival, and tissue repair [75]. However, their dysregulation can facilitate abnormal tissue repair, fibrosis, and the loss of normal growth constraints, which are key features of the early tumor microenvironment [76,77]. Integrin has been reported to be a potential biomarker for an acute kidney injury diagnosis [78]. Its activation in our study also suggests probable renal injury as a result of the GBH exposure. The persistent integrin activation in renal tissues is implicated in glomerulosclerosis and tubulointerstitial fibrosis, both of which are associated with an increased cancer risk [79].

Another important activated pathway in the female cohort is the ribosomal quality control pathway, which helps to eliminate misfolded or defective proteins during protein synthesis [80]. The upregulation of proteins such as proteasome (which is responsible for the degradation of nascent polypeptides) and glyceraldehyde-3-phosphate dehydrogenase and the downregulation of small ribosomal subunit protein eS8 are involved in the prediction of this pathway. The elevation of the ribosomal quality control pathway suggests ongoing translational errors and oxidative stress in the renal tissues as a result of GBH exposure. While the activation of this pathway may serve a protective function, prolonged ribosomal quality control system activation accelerates mutagenesis and genomic instability, which supports tumor growth [81].

The evidence of enhanced inflammatory activity in the GBH-exposed kidneys is further seen through the activation of the neutrophil degranulation pathway. The release of protease and other reactive oxygen species in neutrophil degranulation can cause surrounding tissue damage and perpetuate inflammatory signaling, as observed in acute kidney injuries and chronic kidney disease [82]. This can lead to the damage of kidney cells and an increased susceptibility to infection [83]. The excessive neutrophil degranulation present in renal fibrosis can also lead to the release of neutrophil extracellular traps (NETs), which have been implicated in tumor initiation and progression [84].

Additionally, the activation of the class I MHC antigen presentation pathway may indicate increased immunosurveillance in the renal tissues in response to GBH-induced protein alterations or genomic damage [85]. This process facilitates the recognition of aberrant or damaged cells by cytotoxic T lymphocytes. While this could initially serve as a defense mechanism, the prolonged activation of this pathway may promote immune exhaustion and allow abnormal cells to escape detection, which is an important feature in tumor progression [86].

Furthermore, the chronic GBH exposure in female rats was associated with marked alterations in the expression of key transcriptional regulators within kidney tissues. Notably, transcription factors such as Interleukin-6 (IL-6), the Signal Transducer and Activator of Transcription 3 (STAT3), nuclear factor erythroid 2–like 2 (NFE2L2), and transcriptional enhanced activated domain 1 (TEAD1) were found to be activated, likely contributing to the upregulation of several significant proteins identified in our study (Figure 6b).

IL-6, a well-characterized pro-inflammatory cytokine, plays an important role in tumor progression and metastasis by supporting chronic inflammation and immune evasion [87]. Elevated IL-6 levels have been previously reported in individuals exposed to various pesticides, suggesting a shared inflammatory mechanism underlying chemical-induced toxicity. Its activation in our study reinforces the link between GBH exposure and a tumor-promoting renal microenvironment [88]. Similarly, STAT3—a central mediator of cytokine signaling—was upregulated, highlighting its contribution to cellular processes such as proliferation, resistance to apoptosis, and angiogenesis, all of which are features of cancer [89]. The upregulation of STAT3 has been implicated in both inflammation-driven carcinogenesis and fibrotic kidney disease, suggesting its dual role in mediating GBH-induced renal injury and early neoplastic transformation [90,91].

Additionally, the activation of NFE2L2 (also known as nuclear factor erythroid 2- related factor 2) indicates an adaptive response to the oxidative stress generated by the GBH exposure. NFE2L2 plays a key role in oxidative stress responses by binding to antioxidant response elements, such as detoxifying enzymes, and regulating their expressions [92]. While its activation is initially protective, the persistent or dysregulated activity of NFE2L2 has been shown to facilitate tumor growth by enhancing cellular survival under stress conditions and promoting cellular metabolism changes that support cancer progression [93]. Furthermore, TEAD1, another transcriptional regulator found to be activated in this study, is frequently overexpressed in various cancer cells [94]. It known to contribute to uncontrolled cell proliferation and has been linked to the development of renal fibrosis [95]. The increased expression of TEAD1 suggests that GBH exposure may interfere with normal gene regulation and promote pathological changes in kidney tissues [96].

Some of the implicated diseases and functions in the female cohort include the activation and migration of cells, the cell movement of tumor cell lines, the infection of tumor cell lines, and the inhibition of organismal death. Altogether, the protein expression changes observed in the kidneys of GBH-exposed female rats suggest a coordinated series of molecular disruptions, including the activation of inflammatory pathways, imbalances in protein synthesis and degradation, altered immune responses, and changes in transcriptional regulation. This molecular disruption supports tissue fibrosis, weakens immune surveillance, and ultimately promotes tumorigenesis.

In male rats, only eleven proteins were significantly altered in the GBH-exposed kidneys compared to the control. These changes did not lead to the prediction of any specific pathways or related diseases and functions. However, nucleobindin-1, a calcium-binding protein associated with transcription regulation and immune responses [97], was downregulated, while Ig gamma-1 chain C protein, which plays a role in inflammation [98], was upregulated (Figure 5c). This suggests that an alteration in the protein expression in the male rat’s kidney upon GBH exposure is present but is not large enough to predict significant pathways that can lead to kidney damage or tumors. The absence of overlapping protein changes between sexes further supports a sex-dependent susceptibility to GBH-induced nephrotoxicity, with the female kidneys being more vulnerable to glyphosate-induced oncogenic processes.

A combined analysis of all control and GBH-exposed groups revealed 39 differentially expressed proteins, as depicted in Figure S4. The pathway analysis using the IPA revealed that these changes were primarily associated with disruptions in transcription regulatory pathways and increased inflammation, indicating a systemic cellular response to toxic stress. As illustrated in Figure S5, several key transcriptional regulators, including activating transcription factor 4 (ATF4), cAMP response element-binding protein 1 (CREB1), and angiotensinogen (AGT), were upregulated, while tumor necrosis factor (TNF) was downregulated. Each of these factors plays a critical role in cellular homeostasis, and their dysregulation in response to GBH exposure reveals insights into early molecular changes that may increase the susceptibility of renal tissue to malignancies.

Among the activated transcription regulators, the upregulation of ATF-4 suggests a cellular response to oxidative stress following the GBH exposure and the attempt to restore homeostasis [99]. However, the increased expression of ATF-4 supports cell proliferation and can be hijacked by cancerous cells to sustain rapid tumor growth [100]. Additionally, ATF-4 has been implicated in the development of renal tubulointerstitial fibrosis, a precursor to chronic kidney injury [101].

The cAMP response element-binding protein 1 (CREB1), another transcriptional regulator found to be upregulated, plays a key role at the interface of cellular stress, inflammatory signaling, and transcription regulation. Upon GBH exposure, the elevated CREB1 activity may initially represent a defensive mechanism against cellular stress. However, prolonged activation could promote aberrant gene expression patterns that facilitate tumor formation [102]. Notably, elevated CREB1 levels have been linked to pathways involved in diabetic kidney injury, tubular cell damage from kidney stones, and ischemia–reperfusion injuries, highlighting its relevance in the renal pathology and its potential as a therapeutic target [102,103,104]. Similarly, the upregulation of AGT, a protein that plays a role in the renin–angiotensin system (RAS), suggests a disruption in the pathways that regulate blood pressure, the fluid balance, and electrolyte homeostasis. Elevated AGT levels are associated with hypertension, inflammation, kidney injury, cell migration, and fibrosis, all of which are critical steps in kidney disease progression [105,106].

In contrast, tumor necrosis factor (TNF), a cytokine that regulates inflammation and cell survival, was found to be downregulated [107]. Although elevated levels of TNF are typically associated with acute inflammatory responses, the prolonged inhibition of TNF expression may promote immune evasion as well as sustained angiogenesis, which support oncogenesis [108]. The observed reduction in TNF levels may therefore reflect an adaptive cellular response to reduce excessive proliferation and oxidative damage, though it could inadvertently facilitate tumor progression [109]. Some of the associated diseases and functions observed in the combined analysis include the inhibition of the cell death of epithelial cells and organismal death, as well as the activation of both the cell movement and migration of tumor cells.

The *N*-glycan and proteomics changes observed in this study all point toward increased oxidative stress, inflammation, and carcinogenesis. Therefore, regardless of the agricultural and economic benefits of glyphosate-based herbicides (GBHs), their impact on the health of both mammals and non-mammals is inevitably detrimental. Although the impact may appear subtle, the persistent and cumulative toxicity of GBHs could pose significant long-term risks to humans.

Despite these important findings, our study design presents several limitations that should be acknowledged. This study was conducted on rats, which may not fully replicate the human renal physiology, metabolism, or molecular response. Similarly, although we observed sex-specific effects, the small sample size per cohort may affect the statistical power and generalizability of our study. Additionally, the GBH doses administered may not accurately reflect the typical human exposure levels. Furthermore, our analysis focused primarily on molecular level changes (glycomics and proteomics) without involving a functional assessment of the renal injury, such as histopathological evaluations, or estrous cycle staging for the female rats. These limitations highlight the need for further investigations on a larger scale, to validate and expand upon the molecular insights presented here. Further investigations, including histopathology and renal function tests, are needed to fully define the progression and causal relationship in GBH-induced nephrotoxicity. Such research will be critical for translating these findings into clinically significant strategies for early detection and care in GBH-induced renal dysfunction.

## 5. Perspective for Clinical Practice

Renal dysfunction contributes significantly to the early development of cardiovascular diseases, hypertension, and other systemic complications, which further increase patients’ morbidity and mortality [28,110]. Glyphosate-based herbicides induce aberrant glycosylation patterns and altered protein expression in the kidney, as observed in our study, suggesting molecular disruptions that compromise renal health. Alterations in the *N*-glycan composition, such as increased fucosylation and sialofucosylation, alongside elevated levels of inflammatory and oxidative stress markers (e.g., IL-6, STAT3, NFE2L2) mirror diagnostic markers often seen in various human kidney pathologies [53,111,112]. These changes may represent potential early markers for glyphosate-induced renal impairment.

While further clinical validation is necessary, our findings emphasize the relevance of GBH exposure history in the clinical assessment of kidney health. For populations with a high risk of occupational or dietary exposure, clinicians could consider integrating environmental risk factors into chronic kidney disease screening protocols and personalized management strategies [113]. The early detection of such molecular disruptions could support preventive care and reduce the burden of progressive renal disease.

## 6. Conclusions

This study demonstrates that chronic exposure to glyphosate-based herbicides (GBHs) can induce alterations in *N*-glycan and protein expressions in the kidney, which contribute to oxidative stress, inflammation, and tumor progression. Our findings revealed a greater susceptibility in the female rats, who exhibited a more pronounced shift in both the *N*-glycan abundance and protein expression following the GBH exposure. Clinically, these findings highlight the potential use of glycomic and proteomic alterations as early indicators of renal dysfunctions in populations chronically exposed to GBH. Incorporating these molecular biomarkers into clinical screening protocols may aid in timely detection and more precise clinical management.

Although this study independently investigated changes in the *N*-glycans and protein profile of rat kidneys, further studies integrating glycoproteomic approaches will be essential to fully understand the exact relationship between the altered *N*-glycans and proteins and their involvement in GBH-induced renal toxicity.

## Data Availability

The mass spectrometry data is available at [114]. https://glycopost.glycosmos.org/preview/1508441743682e6743631fc PIN CODE:8470. accessed on 22 May 2025.

## Supplementary Materials

The following supporting information can be downloaded at https://www.mdpi.com/article/10.3390/biom15101399/s1: Figure S1: Extracted ion chromatogram of some Identified *N*-glycan structures. *N*-Glycan structures symbols are denoted as GlcNAc (*N*-acetylglucosamine) , Gal (galactose) , Man(mannose) , Fuc (fucose) , NeuAc (*N*-acetylneuraminic acid) , and NeuGc (*N*-glycolylneuraminic acid) . Figure S2: Representative MS and MS/MS identification process for the *N*-glycans. (a) Extracted ion chromatogram illustrating the core fucosylated *N*-glycan with the composition HexNAc_4_Hex_3_Fuc_1_ (4-3-1-0-0). Inset (b) shows the corresponding full MS spectra for the glycan. Inset (c) shows the tandem MS (MS/MS) spectrum of the HexNAc_4_Hex_3_Fuc_1_ structures, with the key fragment ions annotated adjacent to their respective peaks. *N*-glycan symbols are as in Figure S1. Figure S3: Two dimensional unsupervised principal component analysis (PCA) with a 95% confidence level (a), heatmap of the statistically significant *N*-glycan in the combined analysis of both cohorts. Total control (*n* = 12) vs. total GBH-exposed (*n* = 13) (b), and the boxplot of the two significant *N*-glycans in the combined analysis (c,d). *N*-glycan symbols are as in Figure S1. Figure S4: Two dimensional unsupervised principal component analysis (PCA) with a 95% confidence level (a), and heatmap (b) of the statistically significant proteins in the combined analysis of both cohorts. Total control (*n* = 12) vs. total GBH-exposed (*n* = 13). Figure S5: Summary of the implicated transcription regulators (a) and implicated diseases and functions (b) in the combined analysis of both female and male cohorts. (c) some of the differentially expressed proteins associated with diseases and functions (*—*p* value < 0.05; **—*p* value < 0.01). Table S1: The correlation coefficient (W) and *p*value from Shapiro–Wilk test confirming that the data is normally distributed. Table S2: The relative abundance of statistically significant *N*-glycans between the control and GBH-exposed group in the female cohort, their *p*values and AUC values. *N*-glycan symbols are as in Figure S1 (Note: Combined AUC value =1). Table S3: The relative abundance of statistically significant *N*-glycans between the control and GBH-Exposed group in the male cohort, their *p*values and AUC values. *N*-glycan symbols are as in Figure S1 (Note: Combined AUC value =1). Table S4: The relative abundance of statistically significant proteins in the female cohorts, their corresponding *p*values and AUC values. Table S5: The relative abundance of statistically significant proteins in the male cohorts, their corresponding *p*-values, and AUC values. The ARRIVE guidelines 2.0: Author Checklist.
