# Supplementary material for: Multi-Omics Alterations in Rat Kidneys upon Chronic Glyphosate Exposure
Source: Biomolecules. 2025 Oct 1;15(10):1399. doi: 10.3390/biom15101399 (PMC12564323; doi:10.3390/biom15101399)
Supplement: Supplementary file 1 [file biomolecules-15-01399-s001.zip › biomolecules-3756465-supplementary.pdf]

[SUPPLEMENTARY MATERIAL]

Multi-Omics Alterations in Rat Kidneys Upon Chronic Glyphosate Exposure

Favour Chukwubueze[1], Cristian D. Guiterrez Reyes[1], Jesús Chávez-Reyes[2], Joy Solomon[1], Vishal Sandilya[1], Sarah Sahioun[1], Bruno A. Marichal-Cancino[2] and Yehia Mechref[1*].

[1] Department of Chemistry and Biochemistry, Texas Tech University, Lubbock, Texas.

[2] Department of Physiology and Pharmacology, Center of Basic Science, Autonomous University of Aguascalientes, Aguascalientes, Mexico.

*Corresponding Author

Department of Chemistry and Biochemistry

Texas Tech University

Lubbock, TX 79409-1061

Email: Yehia.Mechref@ttu.edu

Tel: 806-742-3059

Fax: 806-742-1289

Table of Contents

Supplementary Figures:

Figure S1: Extracted Ion Chromatogram of some identified *N*-glycan structures. *N*-Glycan structures symbols are denoted as GlcNAc (*N*-acetylglucosamine), Gal (galactose), Man(mannose), Fuc (fucose), NeuAc (N-acetylneuraminic acid), and NeuGc (*N*-glycolylneuraminic acid).

Figure S2: Representative MS and MS/MS identification process for the *N*-glycans. (a) Extracted Ion Chromatogram illustrating the core fucosylated *N*-glycan with the composition HexNAc$_4$Hex$_3$Fuc$_1$ (4-3-1-0-0). Inset (b) shows the corresponding full MS spectra for the glycan. Inset (c) shows the tandem MS (MS/MS) spectrum of the HexNAc$_4$Hex$_3$Fuc$_1$ structures, with the key fragment ions annotated adjacent to their respective peaks. *N*-glycan symbols are as in Figure S1.

Figure S3: Two dimensional unsupervised principal component analysis (PCA) with a 95% confidence level (a), heatmap of the statistically significant *N*-glycan in the combined analysis of both cohorts. Total Control (n=12) vs total GBH-exposed (n=13) (b), and the boxplot of the two significant *N*-glycans in the combined analysis (c) & (d). *N*-glycan symbols are as in Figure S1.

Figure S4: Two dimensional unsupervised principal component analysis (PCA) with a 95% confidence level (a), and heatmap (b) of the statistically significant proteins in the combined analysis of both cohorts. Total Control (n=12) vs total GBH-exposed (n=13).

Figure S5: Summary of the implicated transcription regulators (a) and implicated diseases and functions (b) in the combined analysis of both female and male cohorts. (c) some of the differentially expressed proteins associated with diseases & functions. (*- p-value < 0.05; **- p-value < 0.01).

Supplementary Tables:

Table S1: The Correlation coefficient (W) and *p*-value from Shapiro-wilk test confirming that the data is normally distributed.

Table S2: The relative abundance of statistically significant *N*-glycans between the control and GBH-Exposed group in the female cohort, their *p*-values and AUC values. *N*-glycan symbols are as in Figure S1. **(Note: Combined AUC value =1)**

Table S3: The relative abundance of statistically significant *N*-glycans between the control and GBH-Exposed group in the male cohort, their *p*-values and AUC values. *N*-glycan symbols are as in Figure S1. **(Note: Combined AUC value =1)**

Table S4: The relative abundance of statistically significant proteins in the female cohorts, their corresponding *p*-values and AUC values.

Table S5: The relative abundance of statistically significant proteins in the male cohorts, their corresponding *p*- values, and AUC values.

The ARRIVE guidelines 2.0: Author Checklist.

Figure S1: Extracted Ion Chromatogram of some identified *N*-glycan structures. *N*-Glycan structures symbols are denoted as GlcNAc (*N*-acetylglucosamine), Gal (galactose), Man(mannose), Fuc (fucose), NeuAc (N-acetylneuraminic acid), and NeuGc (*N*-glycolylneuraminic acid).

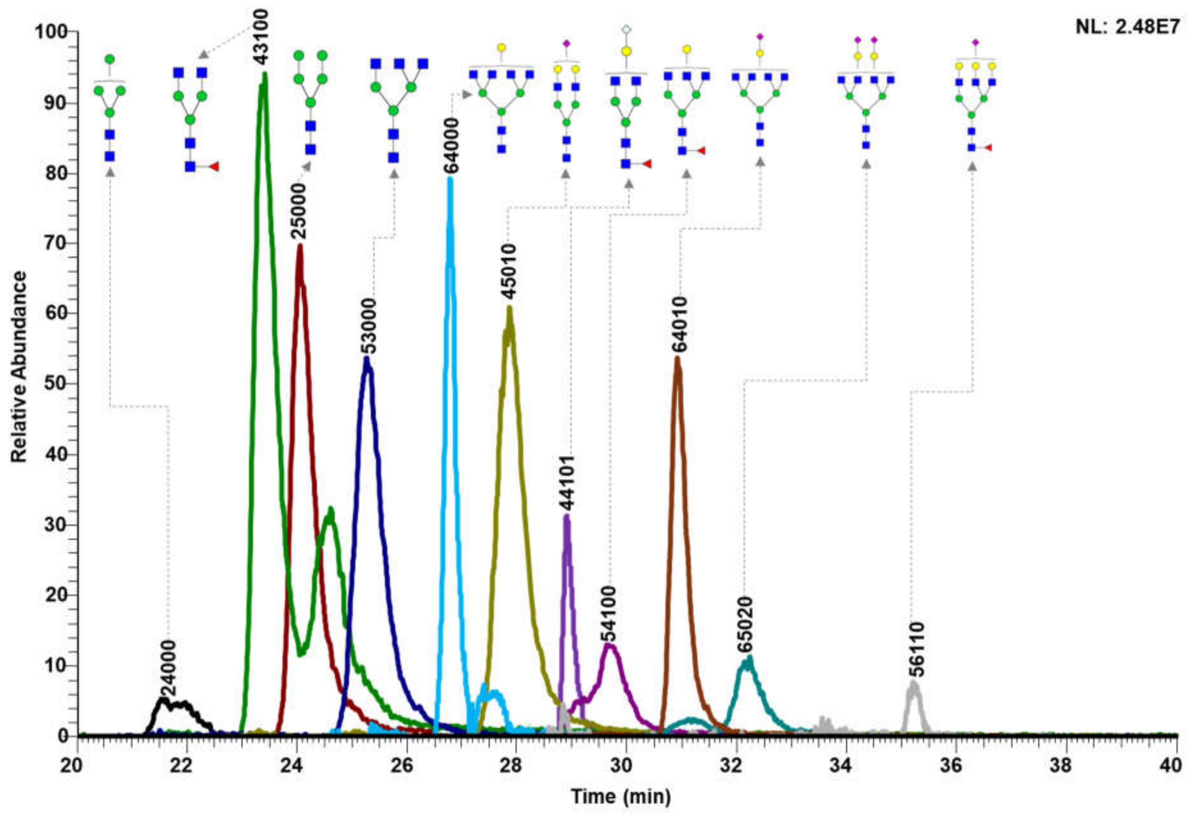

Figure S2: Representative MS and MS/MS identification process for the *N*-glycans. (a) Extracted Ion Chromatogram illustrating the core fucosylated *N*-glycan with the composition $HexNAc_4Hex_3Fuc_1$ (4-3-1-0-0). The inset (b) shows the corresponding full MS spectra for the glycan. The inset (c) shows the tandem MS (MS/MS) spectrum of the $HexNAc_4Hex_3Fuc_1$ structures, with the key fragment ions annotated adjacent to their respective peaks. *N*-glycan symbols are as in Figure S1.

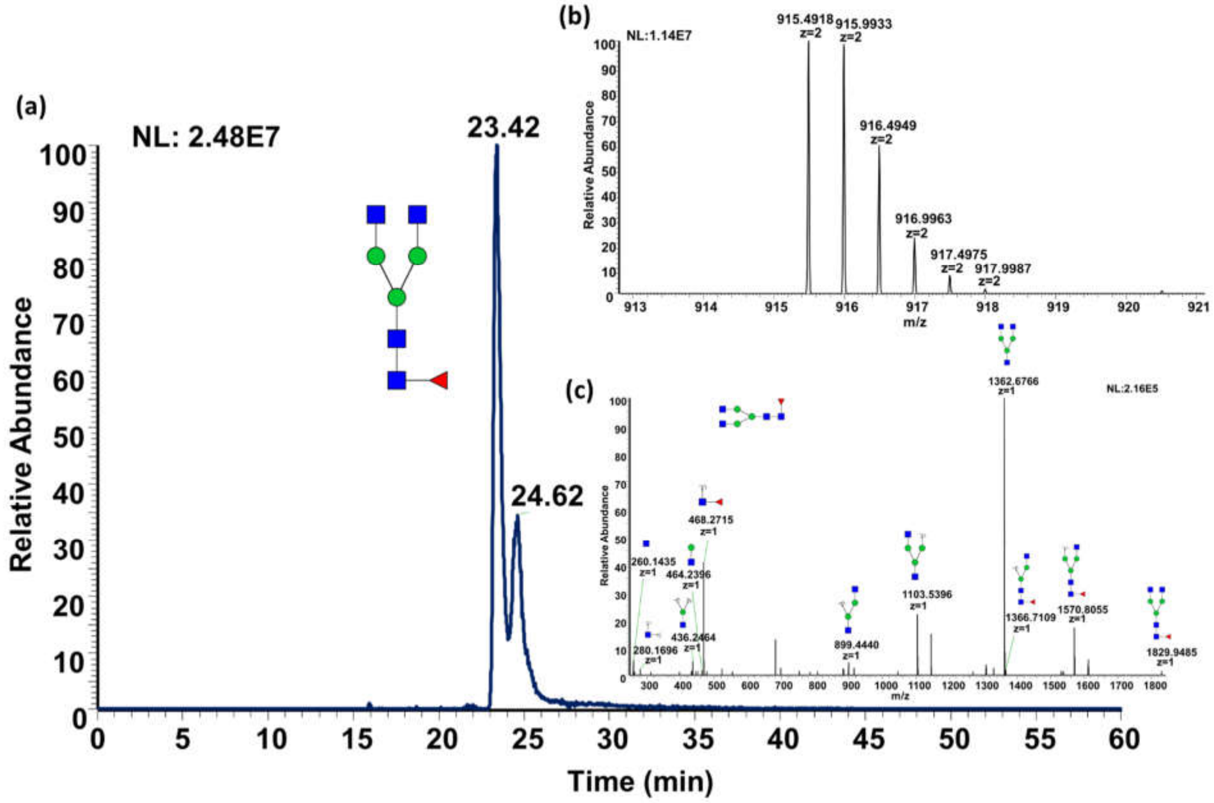

Figure S3: Two dimensional unsupervised principal component analysis (PCA) with a 95% confidence level (a), heatmap of the statistically significant *N*-glycan in the combined analysis of both cohorts. Total Control (n=12) vs total GBH-exposed (n=13) (b), and the boxplot of the two significant *N*-glycans in the combined analysis (c) & (d). *N*-glycan symbols are as in Figure S1.

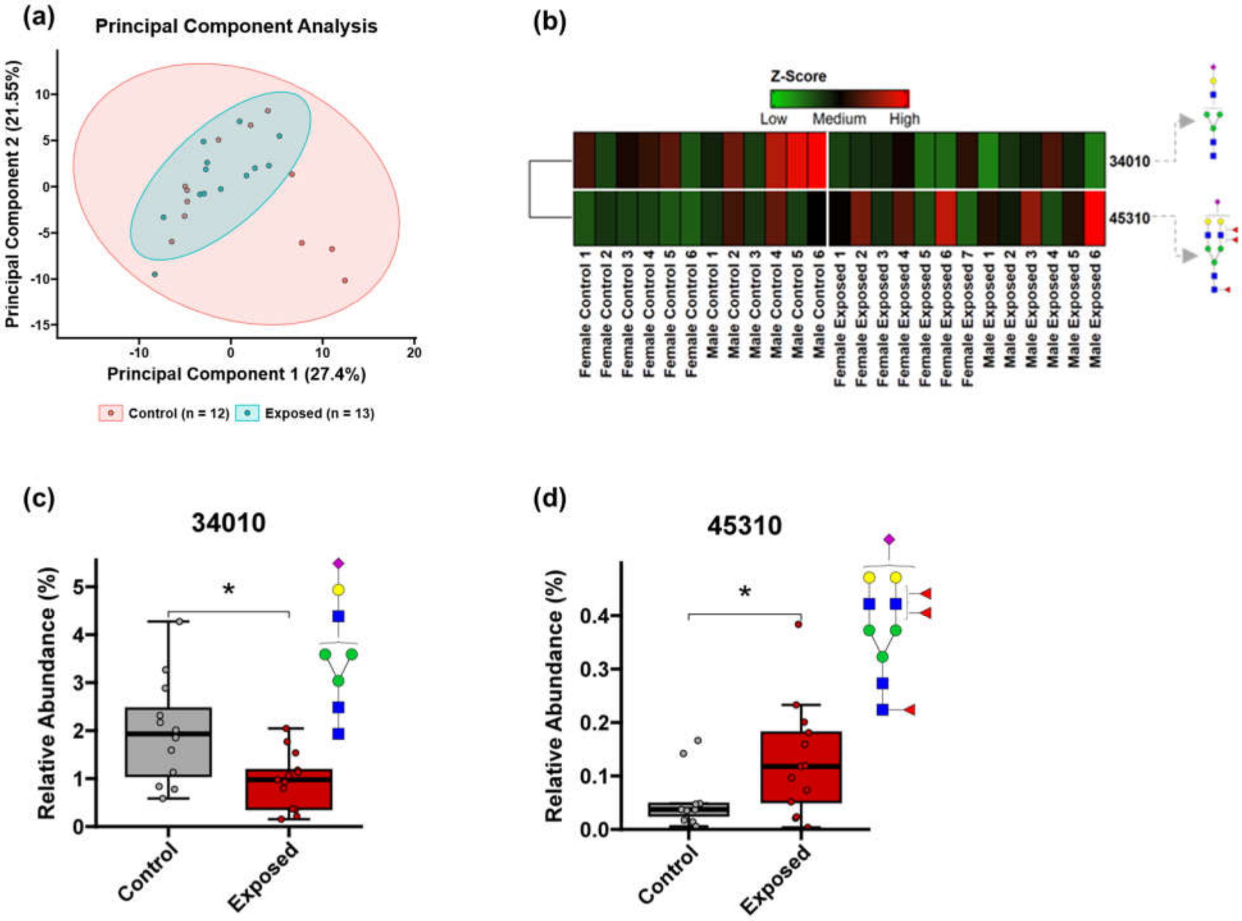

Figure S4: Two dimensional unsupervised principal component analysis (PCA) with a 95% confidence level (a), and heatmap (b) of the statistically significant proteins in the combined analysis of both cohorts. Total Control (n=12) vs total GBH-exposed (n=13).

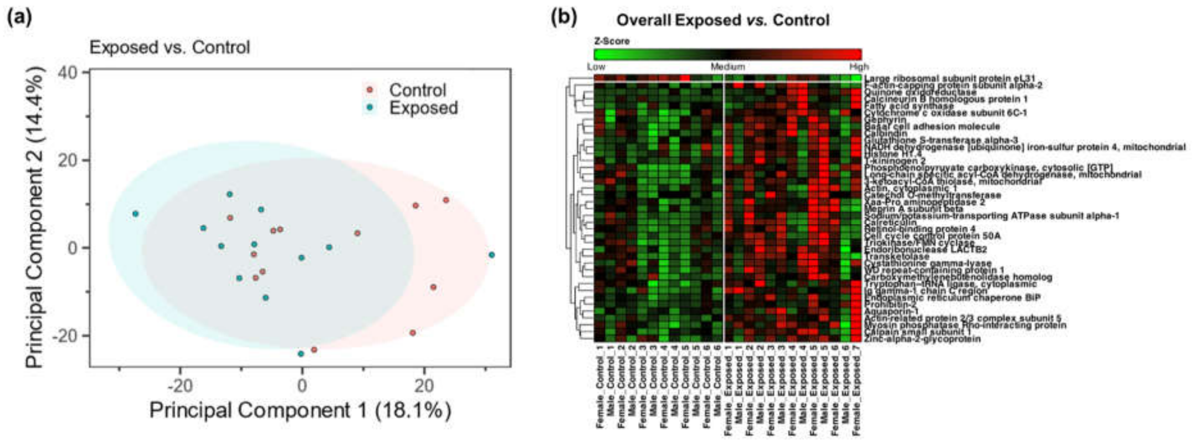

Figure S5: Summary of the implicated transcription regulators (a) and implicated diseases and functions. (b) in the combined analysis of both female and male cohorts. (c) some of the differentially expressed proteins associated with the diseases & functions. (*- p-value < 0.05; **- p-value < 0.01).

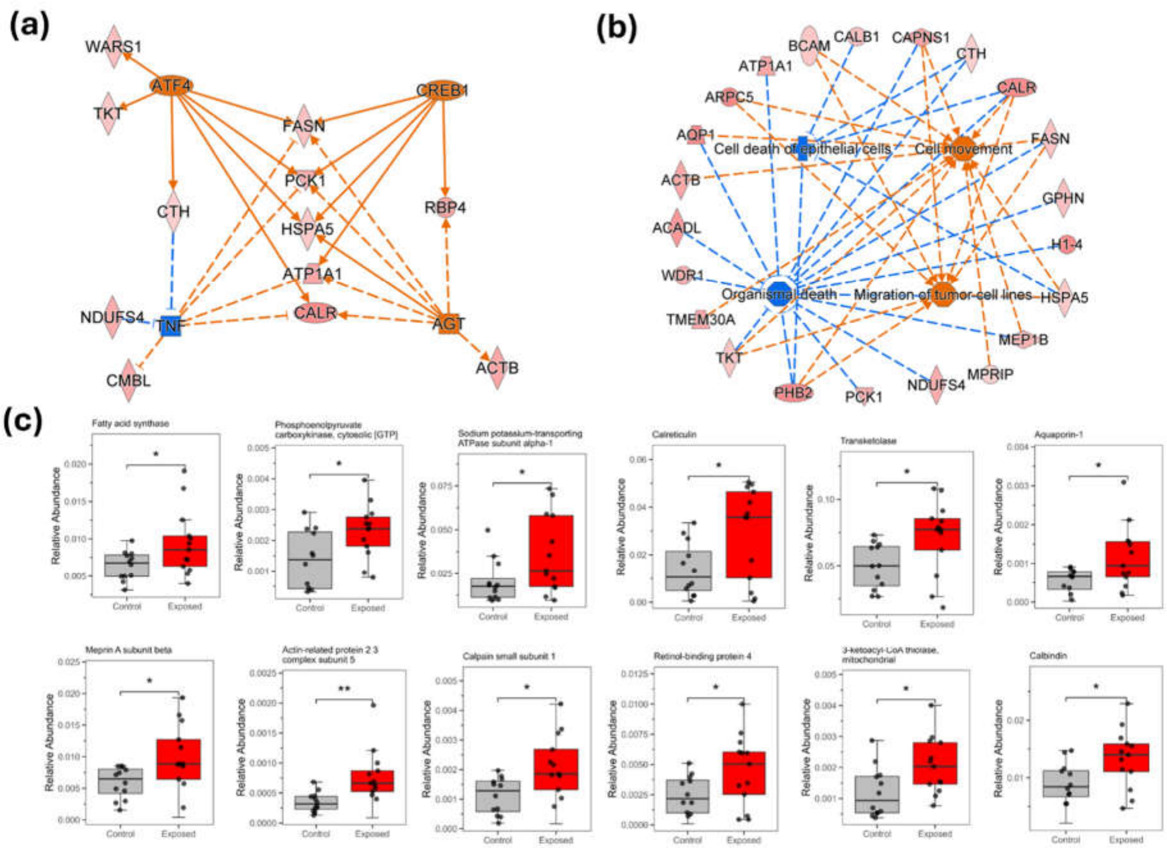

Table S1: The Correlation coefficient (W) and *p*-value from Shapiro-wilk test confirming that the data is normally distributed.

**Cohorts**	**Glycomics**		**Proteomics**	
	**W**	***p*-value**	**W**	***p*-value**
**Male Control**	**0.89**	**0.36**	**0.89**	**0.32**
**Male Exposed**	**0.91**	**0.44**	**0.89**	**0.34**
**Female Control**	**0.88**	**0.28**	**0.89**	**0.29**
**Female Exposed**	**0.91**	**0.41**	**0.90**	**0.31**

Table S2: The relative abundance of statistically significant *N*-glycans between the control and GBH-Exposed group in the female cohort, their *p*-values, and AUC values. *N*-glycan symbols are as in Figure S1. **(Note: Combined AUC value =1)**

***N*-Glycan Composition**	***N*-Glycan Structure**	**Control**	**GBH-Exposed**	***P*- value**	**AUC values**
**68100**	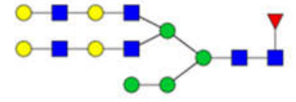	0.22±0.02	0.52±0.02	0.0051	0.93
**77100**	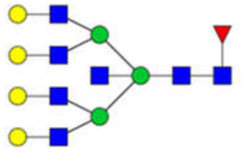	0.32±0.03	0.78±0.05	0.021	0.88
**86100**	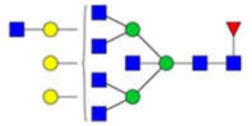	3.4±0.1	5.4±0.2	0.029	0.83
**56100**	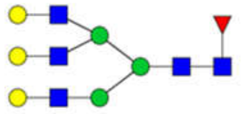	2.9±0.2	5.1±0.2	0.030	0.83
**45210**	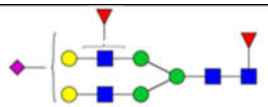	1.41±0.09	4.1±0.3	0.034	0.79
**56000**	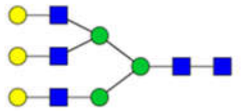	0.42±0.03	0.91±0.05	0.035	0.83
**45300**	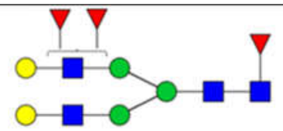	0.33±0.04	1.2±0.1	0.037	0.83

**64100**	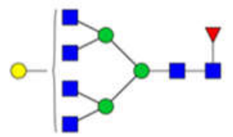	0.38±0.02	0.77±0.04	0.038	0.79
**64000**	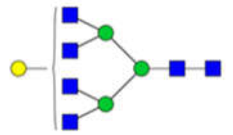	0.16±0.01	0.45±0.04	0.041	0.79
**57100**	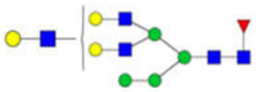	1.4±0.1	2.7±0.1	0.042	0.79
**75110**	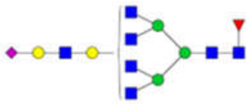	0.18±0.02	0.36±0.02	0.047	0.83
**45310**	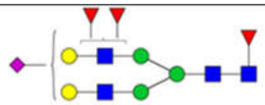	0.16±0.02	0.75±0.1	0.049	0.79

Table S3: The relative abundance of statistically significant *N*-glycans between the control and GBH-Exposed group in the male cohort, their *p*-values and AUC values. *N*-glycan symbols are as in Figure S1. **(Note: Combined AUC value =1)**

***N*-Glycan Composition**	***N*-Glycan Structure**	**Control**	**GBH-Exposed**	***P*- value**	**AUC value**
**25000**	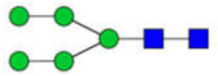	34.0±4	80.0±5	0.014	0.86
**67100**	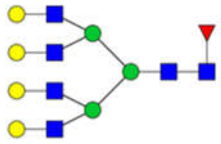	2.8±0.2	1.5±0.1	0.027	0.94
**79100**	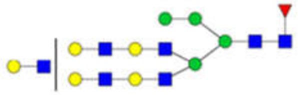	0.23±0.02	0.12±0.01	0.033	0.86
**86100**	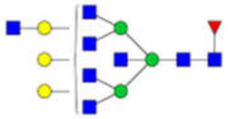	4.2±0.2	2.2±0.2	0.036	0.86
**46101**	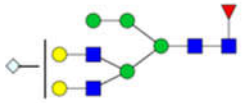	1.8±0.08	0.96±0.1	0.036	0.86
**34010**	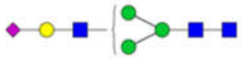	15.0±1	5.6±0.7	0.039	0.81
**23100**	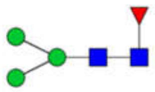	2.3±0.5	6.8±0.7	0.048	0.86

Table S4: The relative abundance of statistically significant proteins in the female cohorts, their corresponding *p*-values and AUC values.

**Proteins**	**Uniprot Accession**	**Control**	**GBH-Exposed**	***P*- value**	**AUC values**
Glyceraldehyde-3-phosphate dehydrogenase, testis-specific	Q9ESV6	0.0091±0.0008	0.023±0.0007	0.00041	1
NADH dehydrogenase [ubiquinone] 1 alpha subcomplex subunit 10, mitochondrial	Q561S0	0.0044±0.0002	0.0089±0.0003	0.0016	0.94
T-kininogen 2	P08932	0.19±0.009	0.29±0.006	0.0056	0.98
Endoribonuclease LACTB2	Q561R9	0.022±0.001	0.033±0.0006	0.0059	0.92
Alpha-2-HS-glycoprotein	P24090	0.016±0.002	0.042±0.01	0.0080	0.90
Microtubule-actin cross-linking factor 1	D3ZHV2	0.32±0.009	0.48±0.007	0.0081	0.93
Alanine--glyoxylate aminotransferase 2, mitochondrial	Q64565	0.033±0.002	0.066±0.002	0.0085	0.90
Succinate dehydrogenase [ubiquinone] flavoprotein subunit, mitochondrial	Q920L2	0.11±0.008	0.22±0.007	0.0088	0.93
Prohibitin 1	P67779	0.015±0.0004	0.030±0.001	0.0099	0.95
Enoyl-CoA delta isomerase 2	Q5XIC0	0.0013±0.0001	0.0035±0.0002	0.0099	0.94
Nibrin	Q9JIL9	0.026±0.001	0.060±0.003	0.011	0.93
Long-chain-fatty-acid--CoA ligase 5	O88813	0.022±0.002	0.048±0.002	0.014	0.88

Tubulointerstitial nephritis antigen-like	Q9EQT5	0.016±0.001	0.029±0.0007	0.015	0.88
Peroxisomal multifunctional enzyme type 2	P97852	0.035±0.002	0.065±0.002	0.017	0.90
Actin, cytoplasmic 1	P60711	0.42±0.03	1.1±0.07	0.018	0.83
Catechol O-methyltransferase	P22734	0.0010±0.0001	0.0036±0.0002	0.018	0.92
Tropomyosin alpha-1 chain	P04692	0.076±0.004	0.14±0.006	0.018	0.86
ADP-ribosylation factor 1	P84079	0.0033±0.0004	0.0067±0.0003	0.019	0.88
Fibrinogen alpha chain	P06399	0.12±0.005	0.18±0.003	0.022	0.90
3-ketoacyl-CoA thiolase, mitochondrial	P13437	0.0069±0.0008	0.016±0.0007	0.022	0.83
Cystathionine gamma-lyase	P18757	0.076±0.006	0.15±0.006	0.024	0.81
Mediator of RNA polymerase II transcription subunit 23	Q5EB59	0.028±0.002	0.048±0.002	0.026	0.88
Endoplasmic reticulum chaperone BiP	P06761	0.25±0.006	0.45±0.02	0.026	0.90
Utrophin	G3V7L1	1.2±0.08	0.62±0.05	0.027	0.83
Protein AMBP	Q64240	0.040±0.001	0.065±0.002	0.028	0.79
Small ribosomal subunit protein uS5	P27952	0.050±0.003	0.075±0.002	0.030	0.86
Calbindin	P07171	0.060±0.004	0.11±0.004	0.030	0.81
Proteasome subunit alpha type-6	P60901	0.052±0.004	0.094±0.003	0.032	0.81
Hsc70-interacting protein	P50503	0.0050±0.0003	0.011±0.0007	0.032	0.81
Dihydropyrimidinase	Q63150	0.020±0.002	0.046±0.002	0.032	0.86
Dehydrogenase/reductase SDR family member 4	Q8VID1	0.0040±0.0002	0.019±0.001	0.034	0.71

Lysozyme C-1	P00697	0.74±0.03	1.5±0.08	0.034	0.81
Xaa-Pro aminopeptidase 2	Q99MA2	0.012±0.001	0.030±0.002	0.034	0.83
Cell cycle control protein 50A	Q6AY41	0.0046±0.0005	0.013±0.001	0.034	0.83
Calnexin	P35565	0.014±0.001	0.040±0.003	0.035	0.86
Zinc-alpha-2-glycoprotein	Q63678	0.0067±0.0007	0.014±0.0007	0.035	0.90
Actin-related protein 2/3 complex subunit 5	Q4KLF8	0.0017±0.0001	0.0058±0.0005	0.036	1
Amino acid transporter heavy chain SLC3A1	Q64319	0.0083±0.0008	0.026±0.002	0.037	0.86
Heterogeneous nuclear ribonucleoprotein F	Q794E4	0.0055±0.0001	0.013±0.0009	0.038	0.83
Small ribosomal subunit protein eS8	P62243	0.0018±0.0001	0.0016±0.0002	0.040	0.82
Calpain small subunit 1	Q64537	0.0071±0.0007	0.017±0.001	0.041	0.86
Cytochrome b-c1 complex subunit 1, mitochondrial	Q68FY0	0.019±0.002	0.053±0.004	0.042	0.76
Haptoglobin	P06866	0.20±0.02	0.36±0.01	0.043	0.88
Sodium/potassium-transporting ATPase subunit alpha-1	P06685	0.095±0.008	0.28±0.02	0.043	0.86
Purine nucleoside phosphorylase	P85973	0.044±0.008	0.097±0.005	0.044	0.86
Protein PRRC1	Q3T1I4	0.038±0.001	0.055±0.001	0.044	0.81
Glutamate--cysteine ligase regulatory subunit	P48508	0.033±0.005	0.11±0.009	0.045	0.81
Ras-related protein Rab-8B	P70550	0.0027±0.0003	0.00094±0.0001	0.045	0.83

Small ribosomal subunit protein RACK1	P63245	0.0071±0.001	0.017±0.001	0.046	0.83
Ribonuclease pancreatic delta-type	Q8VD88	0.0037±0.0004	0.0094±0.0007	0.048	0.80
Meprin A subunit beta	P28826	0.028±0.003	0.076±0.006	0.048	0.81
Elongation factor 1-alpha 2	P62632	0.0050±0.0003	0.0087±0.0004	0.049	0.83
DNA damage-binding protein 1	Q9ESW0	0.028±0.002	0.060±0.004	0.049	0.83

Table S5: The relative abundance of statistically significant proteins in the male cohorts, their corresponding *p*- values and AUC values.

**Protein**	**Uniprot Accession**	**Control**	**GBH-Exposed**	***P* -value**	**AUC values**
Neurolysin, mitochondrial	P42676	0.031±0.002	0.013±0.001	0.0089	0.92
Ig gamma-1 chain C region	P20759	0.050±0.003	0.15±0.01	0.020	1
Cytochrome c oxidase subunit 6C-1	P11950	0.0016±0.0001	0.0029±0.0001	0.024	0.88
Cytosolic purine 5'-nucleotidase	D3ZMY7	0.016±0.0008	0.027±0.001	0.026	0.86
F-actin-capping protein subunit alpha-2	Q3T1K5	0.00074±0.00007	0.0035±0.0003	0.027	0.83
Nucleobindin-1	Q63083	0.030±0.002	0.016±0.0008	0.029	0.94
Tryptophan--tRNA ligase, cytoplasmic	Q6P7B0	0.010±0.0009	0.018±0.001	0.033	0.92
Glycine cleavage system H protein, mitochondrial	Q5I0P2	0.38±0.04	0.13±0.02	0.036	0.86
Urinary protein 2	P81828	0.017±0.001	0.0094±0.0007	0.039	0.83
Large ribosomal subunit protein eL22	P47198	0.0078±0.0006	0.0044±0.0006	0.040	0.77
Myosin regulatory light chain RLC-A	P13832	0.020±0.002	0.037±0.002	0.043	0.86

ARRIVE guidelines 2.0: author checklist

The ARRIVE Essential 10

These items are the basic minimum to include in a manuscript. Without this information, readers and reviewers cannot assess the reliability of the findings.

Item		Recommendation	Section/line number, or reason for not reporting
**Study design**	1	For each experiment, provide brief details of study design including:<br>a. The groups being compared, including control groups. If no control group has been used, the rationale should be stated.<br>b. The experimental unit (e.g. a single animal, litter, or cage of animals).	See section 2.2<br>Line 147- 148
**Sample size**	2	a. Specify the exact number of experimental units allocated to each group, and the total number in each experiment. Also indicate the total number of animals used.<br>b. Explain how the sample size was decided. Provide details of any *a priori* sample size calculation, if done.	See section 2.2, Lines 136 -137, 145- 147<br>Lines 140- 141
**Inclusion and exclusion criteria**	3	a. Describe any criteria used for including and excluding animals (or experimental units) during the experiment, and data points during the analysis. Specify if these criteria were established *a priori.* If no criteria were set, state this explicitly.<br>b. For each experimental group, report any animals, experimental units or data points not included in the analysis and explain why. If there were no exclusions, state so.<br>c. For each analysis, report the exact value of *n* in each experimental group.	Line 143-145<br>None<br>Female control n=6<br>Female exposed n= 7<br>Male control n=6<br>Male exposed n=6
**Randomisation**	4	a. State whether randomisation was used to allocate experimental units to control and treatment groups. If done, provide the method used to generate the randomisation sequence.<br>b. Describe the strategy used to minimise potential confounders such as the order of treatments and measurements, or animal/cage location. If confounders were not controlled, state this explicitly.	Line 143 – 145<br>Line 147 -148
**Blinding**	5	Describe who was aware of the group allocation at the different stages of the experiment (during the allocation, the conduct of the experiment, the outcome assessment, and the data analysis).	Allocation: JCR<br>Exposure protocol: JCR and BAMC<br>Analytical experiments: FC, CDGR, JS, VS and SS<br>Data analysis: FC, VS and CDGR
**Outcome measures**	6	a. Clearly define all outcome measures assessed (e.g. cell death, molecular markers, or behavioural changes).<br>b. For hypothesis-testing studies, specify the primary outcome measure, i.e. the outcome measure that was used to determine the sample size.	Lines 152 -153<br>Lines 152 - 153

**Statistical methods**	7	a. Provide details of the statistical methods used for each analysis, including software used.<br>b. Describe any methods used to assess whether the data met the assumptions of the statistical approach, and what was done if the assumptions were not met.	Section 2.9<br>Lines 318 -319
**Experimental animals**	8	a. Provide species-appropriate details of the animals used, including species, strain and substrain, sex, age or developmental stage, and, if relevant, weight.<br>b. Provide further relevant information on the provenance of animals, health/immune status, genetic modification status, genotype, and any previous procedures.	Section 2.2<br>Not relevant for the experimental protocol
**Experimental procedures**	9	For each experimental group, including controls, describe the procedures in enough detail to allow others to replicate them, including:<br>a. What was done, how it was done and what was used.<br>b. When and how often.<br>c. Where (including detail of any acclimatisation periods).<br>d. Why (provide rationale for procedures).	Section 2 (Materials and methods)
**Results**	10	For each experiment conducted, including independent replications, report:<br>a. Summary/descriptive statistics for each experimental group, with a measure of variability where applicable (e.g. mean and SD, or median and range).<br>b. If applicable, the effect size with a confidence interval.	Tables S2 - S5
